# Percutaneous endoscopic unilateral laminotomy and bilateral decompression under 3D real-time image-guided navigation for spinal stenosis in degenerative lumbar kyphoscoliosis patients: an innovative preliminary study

**DOI:** 10.1186/s12891-020-03745-w

**Published:** 2020-11-10

**Authors:** Tsung-Yu Ho, Chung-Wei Lin, Chien-Chun Chang, Hsien-Te Chen, Yen-Jen Chen, Yuan-Shun Lo, Pan-Hsuan Hsiao, Po-Chen Chen, Chih-Sheng Lin, Hsi-Kai Tsou

**Affiliations:** 1 Department of Orthopedic Surgery, China Medical University Hospital, China Medical University, No. 2, Xueshi Rd., North Dist, Taichung City, 404 Taiwan; 2 Spine Center, China Medical University Hospital, China Medical University, No. 2, Xueshi Rd., North Dist, Taichung City, 404 Taiwan; 3 Biological Science and Technology, National Chiao Tung University, No. 75, Bo’ai St., East Dist, Hsinchu City, 300 Taiwan; 4 Biomedical Science and Engineering, National Chiao Tung University, No. 75, Bo’ai St., East Dist, Hsinchu City, 300 Taiwan; 5 Department of Sports Medicine, College of Health Care, China Medical University, No. 91, Xueshi Rd., North Dist, Taichung City, 404 Taiwan; 6 Department of Orthopedic Surgery, School of Medicine, China Medical University, No. 91, Xueshi Rd., North Dist, Taichung City, 404 Taiwan; 7 Section of Orthopedic Surgery, Department of Surgery, Ministry of Health and Welfare, Changhua Hospital, No. 80, Sec. 2, Zhongzheng Rd., Puxin Township, Changhua County, 513 Taiwan; 8 Functional Neurosurgery Division, Neurological Institute, Taichung Veterans General Hospital, No. 1650, Sec. 4, Taiwan Blvd., Xitun Dist, Taichung City, 407 Taiwan; 9 Department of Rehabilitation, Jen-Teh Junior College of Medicine, Nursing and Management, No. 79-9 Sha-Luen Hu Xi-Zhou Li Hou-Loung Town, Miaoli County, 356 Taiwan

**Keywords:** Navigation, Spinal stenosis, Lumbar spine, Endoscopic surgery, Kyphoscoliosis, Decompression alone

## Abstract

**Background:**

The aim of this study is to introduce a new method of percutaneous endoscopic decompression under 3D real-time image-guided navigation for spinal stenosis in degenerative kyphoscoliosis patients without instability or those who with multiple comorbidities. Decompression alone using endoscope for kyphoscoliosis patient is technical demanding and may result in unnecessary bone destruction leading to further instability. The O-arm/StealthStation system is popular for its ability to provide automated registration with intraoperative, postpositioning computed tomography (CT) which results in superior accuracy in spine surgery.

**Methods:**

In this study, we presented four cases. All patients were over seventy years old female with variable degrees of kyphoscoliosis and multiple comorbidities who could not endure major spine fusion surgery. Percutaneous endoscopic unilateral laminotomy and bilateral decompression under 3D real-time image-guided navigation were successfully performed. Patients’ demographics, image study parameters, and outcome measurements including pre- and post-operative serial Visual analog scale (VAS), and Oswestry Disability Index (ODI) were well documented. The follow-up time was 1 year.

**Results:**

Pre- and post-operative MRI showed average dural sac cross sectional area (DSCSA) improved from 81.62 (range 67.34–89.07) to 153.27 (range 127.96–189.73). Preoperative neurological symptoms including radicular leg pain improved postoperatively. The mean ODI (%) were 85 (range 82.5–90) at initial visit, 35.875 (range 25–51) at 1 month post-operatively, 26.875 (range 22.5–35) at 6 months post-operatively and 22.5 (range 17.5–30) at 12 months post-operatively (*p* < 0.05). The mean VAS score were 9 (range 8–10) at initial visit, 2.25 (range 2–3) at 1 month post-operatively, 1.75 (range 1–2) at 6 months post-operatively and 0.25 (range 0–1) at 12 months post-operatively (*p* < 0.05). There was no surgery-related complication.

**Conclusions:**

To the best of our knowledge, this is the first preliminary study of percutaneous endoscopic laminotomy under O-arm navigation with successful outcomes. The innovative technique may serve as a promising solution in treating spinal stenosis patients with lumbar kyphoscoliosis and multiple comorbidities.

## Background

With aging of the population, spinal stenosis with degenerative kyphoscoliosis has become an increasingly common condition. Lumbar spinal stenosis with or without degenerative spondylolisthesis is a pathologic condition that is often observed in the geriatric population, which is the spinal canal narrowing caused by redundant ligamentum flavum and hypertrophic facet joints and osteophytes, and may present with clinical symptoms such as radicular leg pain or neurogenic claudication [1, 2].

Recent studies have suggested that nonsurgical treatment may not be as effective [3, 4]. The current medical evidence continues to support the role of surgery over non-operative therapies for symptomatic stenosis patients associated with spondylolisthesis [5]. The prospective, randomized, multicenter Spine Patient Outcomes Research Trial (SPORT) also suggests that patients who are treated surgically present with a significantly greater improvement in pain, function, satisfaction, and self-rated progress over 8 years compared to patients treated non-operatively [6–8].

Various studies have evaluated the efficacy of decompression alone versus decompression with fusion for this condition, but the results are still controversial [9–13]. Decompression alone have shown similar results in clinical outcomes compared to decompression with fusion [9, 11, 13]. Open discectomy with laminotomy or laminectomy alone has been considered as standard treatment for lumbar spinal stenosis without instability during the last decades [14]. Percutaneous endoscopic decompression which features minimal amount of tissue injury, less blood loss and a faster recovery serves as a solution to patients with multiple comorbidities. However, decompression alone using endoscope for kyphoscoliosis patient is technically demanding and may result in unnecessary bone destruction leading to iatrogenic instability [15, 16].

Considerable advances in 3D real-time image navigation have changed the nature of spine surgery [17, 18]. The advantages with the use of 3D real-time image navigation include multiple level images in a single sequence and accuracy [19]. With the assistance of 3D real-time image navigation, the depth and location of endoscope trocar, high speed burr and relevant anatomy could be instantly demonstrated on the monitor during the procedure. The amount of bony structure removed could be well-visualized on the monitor. Thus, the risk of inadequate decompression and unnecessary bone destruction with iatrogenic instability could be thus limited. This is the first study reporting endoscopic decompression surgery under 3D real-time image navigation to treat degenerative kyphoscoliosis patients.

The purpose of this study is to describe a novel method which combined full-endoscopic spinal decompression and 3D real-time image navigation. The preliminary result shows adequate and precise decompression without leading to iatrogenic instability when treating spinal stenosis in degenerative kyphoscoliosis patients.

## Methods

### Patient enrollment

**Fig. 1:**
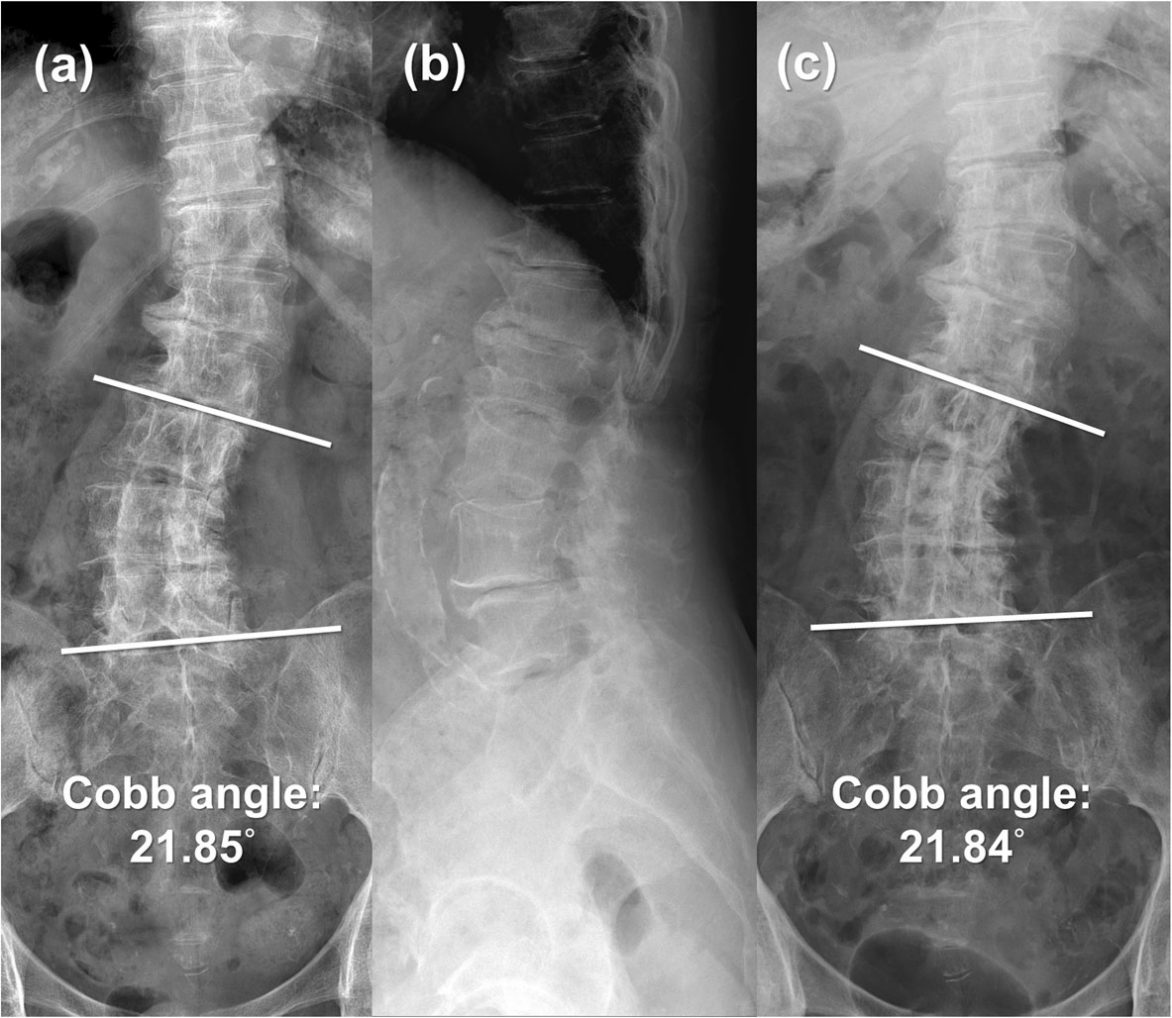
**a** Preoperative AP X-ray shows 21.85° Cobb angle scoliosis from L3-L5. **b** Preoperative lateral X-ray reveals mild spondylolisthesis over L4-L5. **c** Postoperative AP X-ray shows 21.84° Cobb angle scoliosis from L3-L5. **a**, **c** Serial images demonstrate no progression of the scoliosis

Four kyphoscoliosis patients diagnosed with lumbar spinal stenosis between March 2017 and January 2018 were enrolled in this study (Fig. 1). Our inclusion criteria were as follows: lumbar central or lateral recess spinal stenosis with kyphoscoliosis, radicular leg pain or claudication refractory to conservative treatment for at least 6 months, absence of spinal instability confirmed by dynamic radiographs. Instability is considered as of sagittal angulation values > 15°, values in millimeters of translation on the sagittal plane (or coronal plane) > 4 mm or shift > 15% of the inferior vertebral endplate measure. We excluded patients with lumbar foraminal spinal stenosis, spondylolisthesis, compression or burst fracture, infection, tumor, previous lumbar surgery. The participants included four women with an average age of 80.25 years (range, 73–86 years). The average Body Mass Index (BMI) was 24.58 (range 19.94–28.36). The affected levels ranged from L2-S1. The operation level was decided according to clinical presentation and MRI findings. As for the degree of spinal deformity, data were summarized in Table 1 According to Schwab Classification [20].

**Table 1:** Profiles of Patient with Kyphoscoliosis

	Case 1	Case 2	Case 3	Case 4
Age (years)	73	86	82	80
BMI (kg/m^2^)	28.36	23.37	19.94	26.63
Comorbidities	Diabetes Mellitus Chronic Kidney Disease StageIII Chronic Obstructive Pulmonary Disease	Paroxysmal atrial fibrillation Hypertension Diabetes Mellitus Chronic Kidney Disease StageII	Chronic Kidney Disease StageIII Dementia	Hypertension Diabetes Mellitus Congestive Heart Failure
Operation Time (minutes)	272	315	154	233
Schwab Classification for Adult Spinal Deformity				
Type and Location of Deformity	TypeV Lumbar Major Curve	TypeV Lumbar Major Curve	TypeV Lumbar Major Curve	TypeV Lumbar Major Curve
Lordosis Modifier: Sagittal Cobb angle from T12 to S1	20.36 degrees	20.31 degrees	21.14 degrees	21.6 degrees
Subluxation Modifier: Frontal or Sagittal Plane (Anterior or Posterior), Maximum Value	++ Frontal subluxation 16 mm (> 7 mm)	+Frontal subluxation 6 mm (1-6 mm)	++ Frontal subluxation 9.4 mm (> 7 mm)	+ Frontal subluxation 5.53 mm (1-6 mm)
Global Balance Modifier: Sagittal Offset from Posterior Superior Corner S1	Positive: 7.98 cm	Positive: 7.13 cm	Positive: 7.01 cm	Positive: 4.63 cm

### Outcome evaluation

**Fig. 2:**
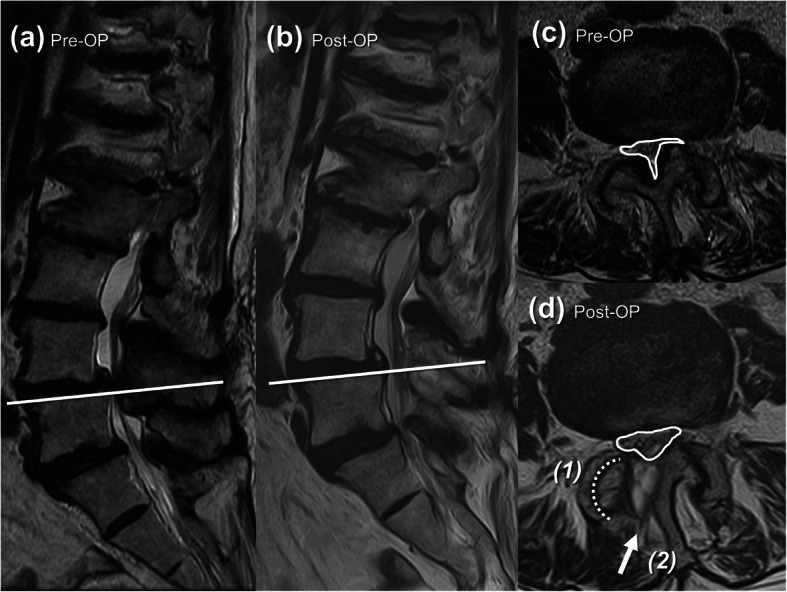
**a** Preoperative sagittal section of T2WI MRI shows severe stenosis over L4-L5. **b** Postoperative sagittal section of T2WI MRI shows stenosis being relieved with intact posterior elements. The decompression level (White line) on axial section of T2WI MRI is shown in (**c**) and (**d**). Comparing **c** preoperative and **d** postoperative T2WI axial section MRI, dura sac cross-sectional area (DSCSA) increases significantly after endoscopic laminotomy at L4–5 under O-arm navigation (1) without any facet joints damage, via (2) interlaminar approach

Radiology outcome measurement including serial radiographs (Fig. 1) and dural sac cross sectional area (DSCSA) on MRI were well documented (Fig. 2) (Table 2). As for the clinical outcome evaluation, the Oswestry Disability index (%) (ODI), Visual analogue scale (VAS) were collected at the initial visit and at 1 month, 6 months and 12 months post-operatively (Table 3).

**Table 2:** Patient Radiology Results

	Operation Level	Pre-OP DSCSA (mm^2^)	Post-OP DSCSA (mm^2^)
**Case 1**	L3/L4	89.07	189.73
**Case 2**	L4/L5	84.39	143.05
**Case 3**	L2/L3	85.68	152.34
**Case 4**	L3/L4	67.34	127.96

*DSCSA* Dural Sac Cross Sectional Area, *Pre-OP* Preoperative, *Post-OP* Postoperative

**Table 3:** Patient Clinical Results

	Diagnosis	Pre-OP		Post-OP					
		ODI	VAS	ODI 1 Month	VAS 1 Month	ODI 6 Month	VAS 6 Month	ODI 12 Month	VAS 12 Month
**Case 1**	Kyphoscoliosis with Spinal Stenosis L3-L4	87.5	9	25	3	22.5	1	17.5	1
**Case 2**	Kyphoscoliosis with Spinal Stenosis L3/L4/L5	82.5	10	35	2	25	2	20	0
**Case 3**	Kyphoscoliosis with Spinal Stenosis L2/L3/L4	90	9	51	2	25	2	22.5	0
**Case 4**	Kyphoscoliosis with Spinal Stenosis L3/L4	80	8	32.5	2	35	2	30	0

*VAS* Visual Analogue Scale, *ODI* Oswestry Disability index, *Pre-OP* Preoperative, *Post-OP* Postoperative

### Statistical analyses

Statistical Analyses were performed using SPSS for Windows, version 24 (SPSS Inc., Armonk, NY). The Friedman two-way analysis of variance (ANOVA) by ranks test was used as a non-parametric test. A *p* < 0.05 was considered statistically significant.

### Navigation system and instruments

The O-arm/StealthStation system (Medtronic Inc., Minneapolis, MN, USA), a 3D real-time image-guided navigation system, is popular for its ability to provide automated registration with intraoperative, postpositioning computed tomography (CT). With SureTrak® II Universal Tracker (Medtronic Inc., Minneapolis, MN, USA) attached to the endoscope instrument, the depth and position of the endoscopic working channel could be observed in real-time fashion on the O-arm/StealthStation monitor.

The Vertebris® Spine Endoscope system (Richard and Wolf, Knittlingen, Germany) features high-resolution endoscope with a 6.9 × 5.6 mm diameter and a 4.1 mm intra-endoscopic working channel. The angle of vision is 25°. The working sleeve has an 8.0 mm outer diameter and a beveled opening, which enable visual and working fields creation in an area with a clear, anatomically preformed cavity.

Surgitron, a high-voltage bipolar probe (Ellman Innovations, New York, USA), is well-known for pinpoint coagulation in a wet field with simultaneously minimal burning or charring of soft tissue. The thermocoagulation device aims to maximized hemorrhage control and thus improves the visibility of the operative field.

### Surgical technique

#### Patient preparation

The surgery was conducted under general anesthesia. The patient was positioned prone on a well cushioned and supportive radiolucent table with the abdomen hanging free. Bilateral knees were flexed to over 90 degrees as possible for the opening of interlaminar space. Back skin was then well prepared and draped.

#### Reference pin insertion and image acquisition

First, we made a small incision over the iliac crest. We inserted the reference pin through the cannula, and used an impactor to nail the pin into the bone until the tap cap bottoms out on the cannula. Then we removed the tap cap and cannula from the pin, placed the spine reference on the pin and rotated the assembly to lock the frame in place.

The spinal segment of interest was scanned using the O-arm navigation and the images were automatically registered to the Stealth Station. All the navigational instruments were registered.

#### 3D real-time image-navigated percutaneous endoscopic decompression

**Fig. 3:**
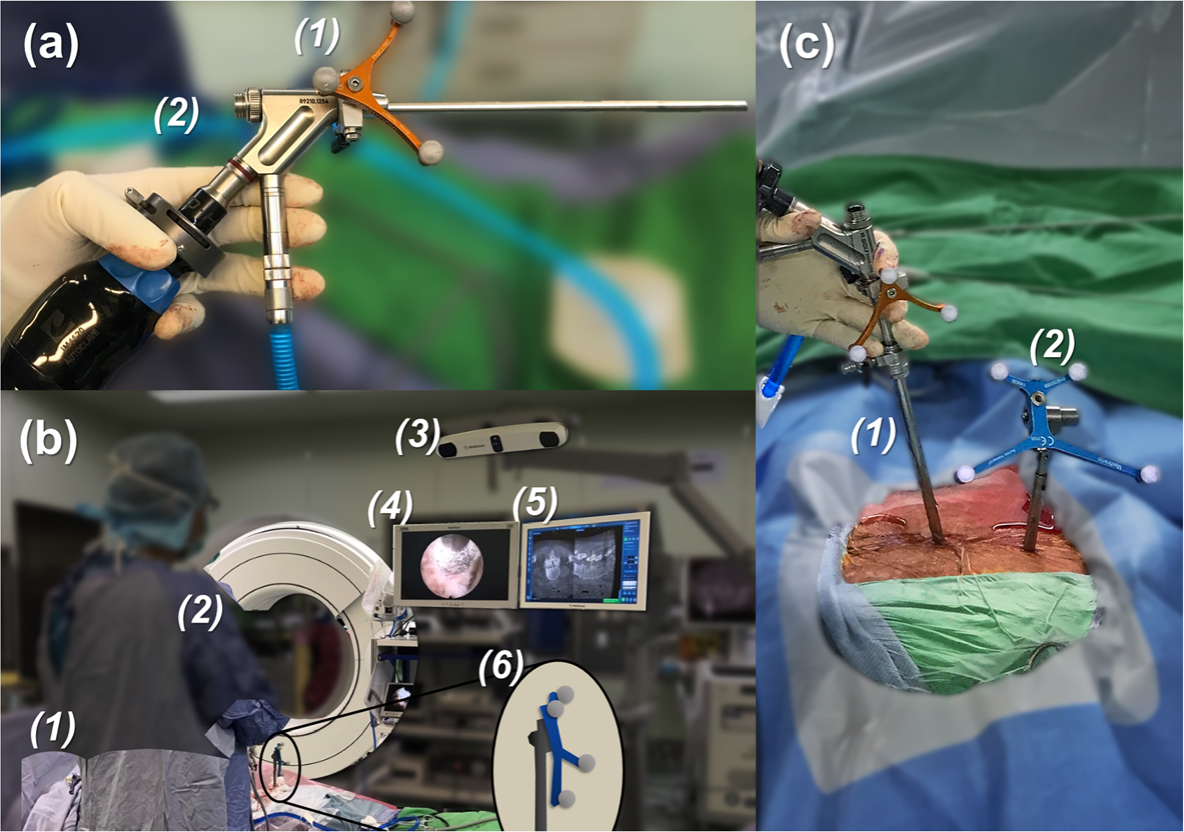
**a** Intraoperative photo shows the attachment of (1) SureTrak® II Universal Tracker, Small Passive Fighter to the (2) Vertebris® Spine Endoscope. **b** Photos reveals the setting of surgery, including (1) patient positioning, (2) (3) O-arm setting, (4) endoscope monitoring, (5) navigation monitoring and (6) reference pin position. (c) Photo shows the relative position of (1) the endoscope in the sleeve, and (2) the percutaneous reference pin (in the area over the iliac crest)

Interlaminar approach was chosen. The skin incision was made under O-arm navigation, which was under the spinal laminal junction. A dilator, 8.0 mm in outer diameter, was bluntly inserted to the edge of the interlaminar window. Then, an operative sleeve with a beveled opening was directed towards the ligamentum flavum. After attaching the SureTrak® II Universal Tracker to the Vertebris® Spine Endoscope (Fig. 3), the endoscope was inserted into the working channel (Fig. 3).

**Fig. 4:**
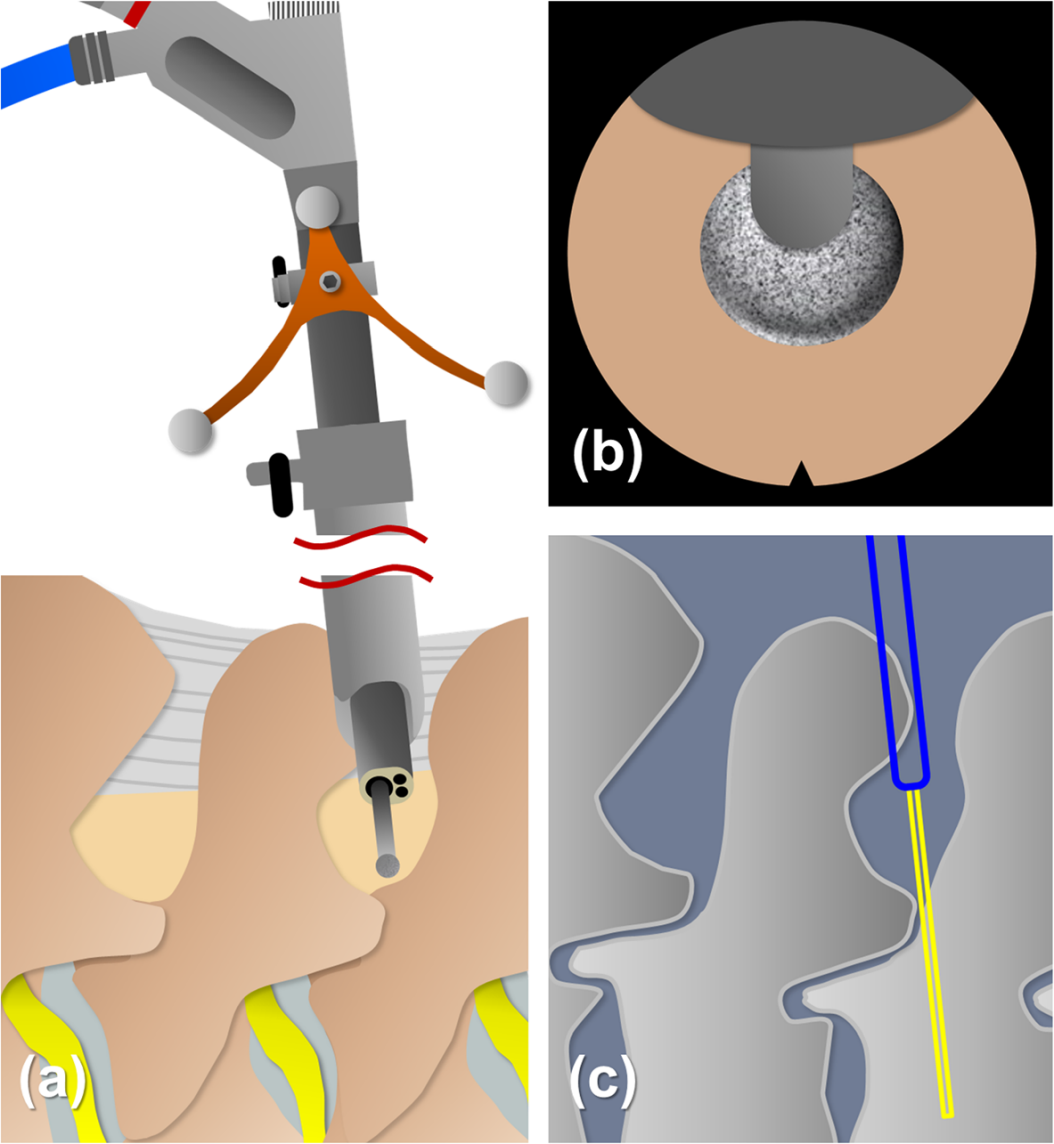
Pattern diagram shows (**a**) the navigational instrument set-up with universal tracker attached to the endoscope. The burr tip docks on the lamina. **b**, **c** The depth of the endoscope in the working field can be simultaneously seen on the navigation monitor. The blue bar indicates the tip of endoscope and the yellow bar points out the trajectory of burr

**Fig. 5:**
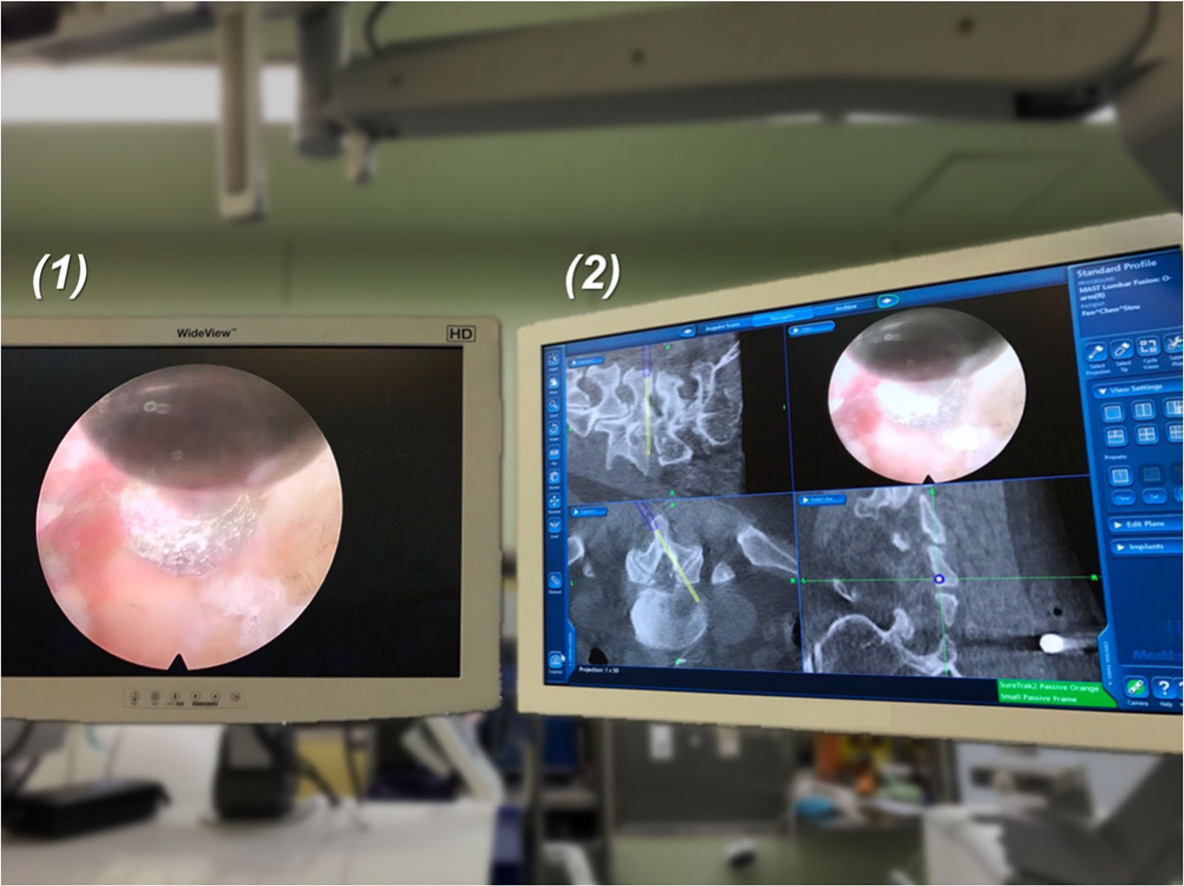
Combining (1) Endoscopic images and (2) O-arm navigation images, the trajectory of the burr is navigated. The depth of burr could be adjusted precisely during laminotomy under 3D real-time O-arm navigation

**Fig. 6:**
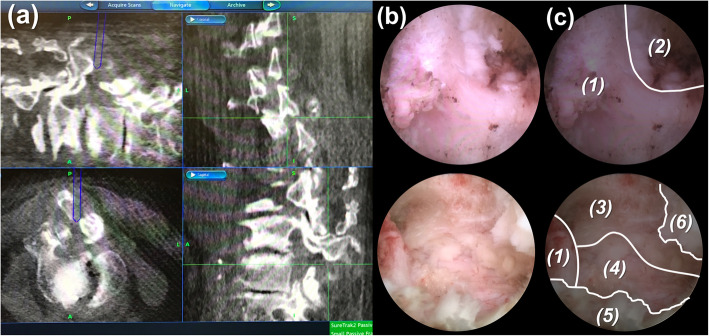
Surgeon performs endoscopic laminotomy under precise O-arm navigation. Intraoperative photos show **a** O-arm navigation images **b** Endoscopic images and **c** its introduction: (1) base of spinous process, (2) interlaminar space, (3) right side lateral recess, (4) dura, (5) left side lateral recess, (6) ligamentum flavum

After introduction of the endoscope, the bony boundaries of superior lamina, inferior lamina and facet joint were identified and the soft tissues were removed with bipolar probe, punch and forceps. To broaden the working space, a minimal bone resection was made from medial to lateral. 3-4 mm of bone around superior and inferior lamina was removed with a diamond burr. Then, the approach proceeded to the superior articular process, creating space for lateral recess decompression. The depth and location of the bony landmark could be well visualized through the O-arm navigation system (Figs. 4 and 5). The O-arm navigation system offered the real-time images as a warning sign before the facet joint was violated, which prevented the spinal column from instability. Not until the cranial, caudal, medial, and lateral bony boundaries of the operative field were well prepared should the ligamentum flavum being opened. A lateral window of approximately 4–6 mm was made on the ligamentum flavum. The neural structures and epidural fat tissues were exposed. The perineural membrane was dissected from the neural structure carefully under direct endoscopic visualization. The operating sleeve with beveled opening could be turned and used as a nerve hook. With the joystick technique, the hypertrophic ligamentum flavum could be removed by controlling endoscope in either direction. The contralateral lateral recess decompression was achieved through unilateral approach with bilateral decompression technique. Finally, epidural bleeding was checked and well-controlled by Surgitron bipolar probe. The whole procedure was done safely and effectively under precise O-arm navigation (Fig. 6).

## Results

### Hospital course

**Fig. 7:**
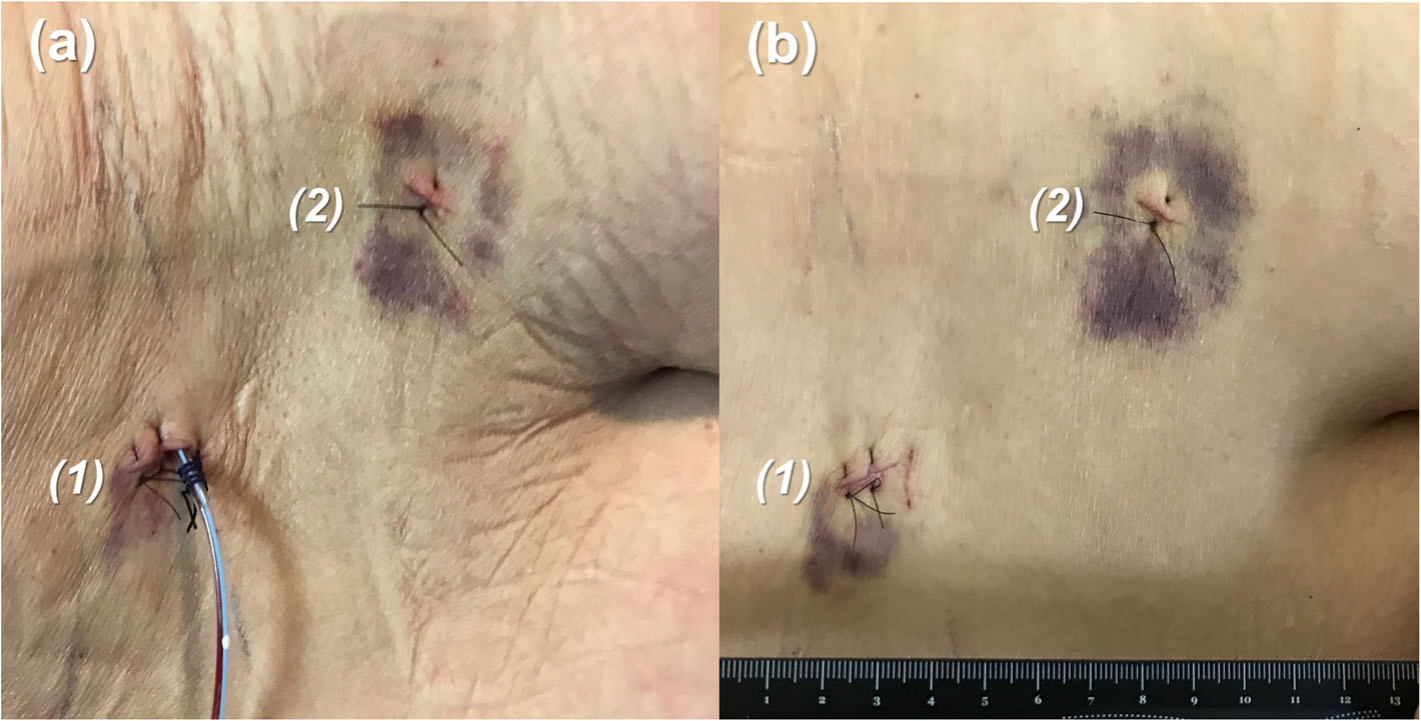
**a** Surgical wounds on postoperative day 1 with a hemovac drainage in the endoscope insertion wound; **b** Both (1) the endoscope insertion wound and (2) the percutaneous reference pin insertion wound are about 1 cm

Without unnecessary bone destruction, the spinal stenosis was successfully decompressed through endoscopic surgery. Average operation time was 243.5 min (range 154–315 min). Both the endoscopic insertion wound and the reference pin insertion wound were about 1 cm (Fig. 7). All patients could stand and walk freely on a walker on postoperative day 1. The hospitalization time was within 3 days.

### Radiology results

The efficacy of decompression was confirmed by MRI at 12 months follow-up post-operatively. Average dural sac cross sectional area (DSCSA) improved from 81.62 (range 67.34–89.07) to 153.27 (range 127.96–189.73) (Fig. 2) (Table 2). The follow-up X-ray performed at 12 months post-operatively showed no obvious Cobb angle change which indicated no progress of the scoliosis (Fig. 1).

### Clinical results

As shown in Table 3, the mean ODI (%) were 85 (range 82.5–90) at initial visit, 35.875 (range 25–51) at 1 month post-operatively, 26.875 (range 22.5–35) at 6 months post-operatively and 22.5 (range 17.5–30) at 12 months post-operatively (*p* < 0.05). The mean VAS score were 9 (range 8–10) at initial visit, 2.25 (range 2–3) at 1 month post-operatively, 1.75 (range 1–2) at 6 months post-operatively and 0.25 (range 0–1) at 12 months post-operatively (*p* < 0.05). There was no surgery-related complication such as inadequate decompression, dural tear, iatrogenic neurological injury, substantial blood loss unnecessary bone destruction with iatrogenic instability.

## Discussion

To the best of our knowledge, this is the first clinical study utilizing the technique of percutaneous endoscope with O-arm navigation to treat spinal stenosis for kyphoscoliosis patients. The advantages of this combined technique are high precision with promising decompression effect, minimal invasive surgery with little soft tissue damage, and preservation of original spinal stability.

Recent studies have demonstrated that in patients whose primary complain are radiculopathy with an underlying biomechanically stable spine, decompression surgery alone with a less invasive technique may be sufficient. Decompression with fusion does not appear to be more effective than decompression alone when considering pain relief, walking ability, or disability status [21, 22].

The gold standard method of the decompression of lumbar spinal stenosis is laminectomy with or without lateral recess and foraminal decompression. A laminectomy removes the entire lamina and the underlying ligamentum flavum, while a laminotomy removes only a small bone window from the lamina, unilaterally or bilaterally, which could be accomplished through minimally invasive surgery, such as percutaneous endoscope technique [23, 24]. Minimally invasive decompression plays an important role for elderly patients. For elderly patients with focal lumbar spinal stenosis, outcomes and surgical morbidity have been shown to be similar to those in the younger demographics [25].

Several studies have focused on the efficacy of percutaneous endoscopic surgery for spinal stenosis. Ito et al. demonstrated improved VAS, ODI and Japanese Orthopedic Association (JOA) score after conducting endoscopic decompression with sublaminar approach [26]. Kang et al. compared biportal endoscopic surgery with microscopic surgery and found that there was shorter hospitalization time, shorter operation time, less hemovac drainage and less opioid use in endoscopic surgery group [27]. Kim and Choi found satisfactory results at minimum 2-year follow-up with no wound infection or segmental spinal instability after conducting endoscopic decompression for lumbar spinal stenosis [28]. Qin et al. reported there was lower access time with navigated percutaneous endoscopic lumbar discectomy compared with conventional techniques [29]. Comparative analysis between microscopic, tubular and endoscopic decompression also discovered that tubular and endoscopic surgery group showed less invasiveness with less increase of serum CPK enzyme level, shorter hospitalization time, and less immediate postoperative back pain [30]. The efficacy and safety of endoscopic decompression have been thoroughly investigated.

The original treatment plan for our patients was open surgery and correction of scoliosis with long fusion to sacrum (in order to avoid fusion above the apex of curve) with laminectomy. However, the adverse effect of long fusion brings certain early perioperative complications in old age patients with multiple comorbidities [31]. Therefore, decompression alone using percutaneous endoscope seemed to be a reasonable alternative. Decompression alone with laminectomy may lead to iatrogenic instability. However, with accurate O-arm guided navigated decompression, excessive bone removal could be successfully avoided. The O-arm navigation monitor could give a warning image before the facet joint is violated. With this combined technique, it was possible to overcome abnormal anatomical challenges [32, 33]. Decompression alone in old patients with multiple comorbidities was difficult to achieve adequate decompression without unnecessary bony destruction. O-arm navigation provided a new option for this kind of patients. With O-arm navigation, this technique could be applied in even more complicated cases, particularly in the elderly or immunocompromised patients, as well as in patients with multiple comorbidities [34].

However, the technique still carries some disadvantages. To begin with, additional wound is needed for reference pin placement. Then, there is prolonged anesthesia and surgical time due to individual surgical instrument registration with the navigation system. Furthermore, it is essential to assure the reference frame not being bumped to avoid navigation inaccuracy. The need to keep the tracking tools in line with the navigation system, will incur some restraints on the surgeon’s movements during decompression steps thus further adding to surgical time. Finally, operative manipulation of endoscope and relevant instruments requires delicate surgical skills due to the limited operative field.

The present study has some limitations. For one thing, there are a limited number of patients with no control group for comparison. For another, the follow-up is relative short, lacking long-term follow-up data. However, the purpose of this study is to describe the combined technique and assess its clinical and radiological results. Decompression alone using endoscope under O-arm navigation could be considered in patients with multiple comorbidities who could not endure major surgery and surgery-related hazardous complication.

## Conclusions

To the best of our knowledge, this is the first preliminary study of percutaneous endoscopic laminotomy under O-arm navigation with successful outcomes. Percutaneous endoscopic laminotomy under O-arm navigation is a minimal invasive procedure compared to open posterior surgery, such as decompression surgery plus fusion surgery, with no excessive bone and soft tissue destruction, smaller cosmetic wound, shorter hospitalized duration, but similar treatment effects. We believe this novel technique is promising for spinal stenosis patients with lumbar kyphoscoliosis and multiple comorbidities. Further studies on larger sample are required to support these preliminary results.

## Data Availability

The datasets used and analyzed during the current study are available from the corresponding author on reasonable request.

## Abbreviations

BMI: Body Mass Index
3D: Three-Dimensional
AP: Anteroposterior
CT: Computed Tomography
MRI: Magnetic Resonance Imaging
T2WI: T2-Weighted Image
ODI: Oswestry Disability Index (%)
VAS: Visual Analogue Scale
JOA: Japanese Orthopedic Association
